# Obstructive Jaundice Due to Periportal Tuberculous Lymphadenopathy in a Patient With Multidrug-Resistant (MDR) Tuberculosis: A Case Report

**DOI:** 10.7759/cureus.107124

**Published:** 2026-04-15

**Authors:** Ayush Mali, Anil Sontakke, Saood Ali

**Affiliations:** 1 Respiratory Medicine, NKP Salve Institute of Medical Sciences, Nagpur, IND

**Keywords:** lymph node tuberculosis, mdr tuberculosis, obstructive jaundice, porta hepatis, ptbd, rise and fall phenomenon

## Abstract

Extrapulmonary tuberculosis is a common manifestation of tuberculosis, with lymph node involvement being the most frequent form. Among its presentations, periportal lymph node involvement leading to obstructive jaundice is extremely uncommon. Jaundice developing during anti-tubercular therapy (ATT) is frequently ascribed to drug-induced hepatotoxicity, which may contribute to delays in accurate diagnosis. We report a 17-year-old male with multidrug-resistant (MDR) lymph node tuberculosis who presented with progressive jaundice and right upper quadrant abdominal pain. Laboratory evaluation showed marked conjugated hyperbilirubinemia and cholestatic enzyme elevation. Ultrasonography, contrast-enhanced computed tomography (CECT), and magnetic resonance cholangiopancreatography (MRCP) revealed a large periportal and peripancreatic coalescent lymph nodal mass compressing the common bile duct (CBD), causing upstream biliary dilatation. Differential diagnoses included drug-induced hepatitis and cholangiocarcinoma, but radiology favored tuberculous lymphadenitis. The patient underwent percutaneous transhepatic biliary drainage (PTBD), with significant clinical improvement. He was subsequently restarted on the all-oral longer MDR regimen under careful monitoring. Obstructive jaundice due to tuberculous lymphadenopathy is a rare but important entity that may mimic drug-induced hepatitis or malignancy. Early imaging, biliary decompression, and individualized ATT modification are crucial. This case also highlights the rise and fall pattern associated with evolving drug resistance.

## Introduction

Tuberculosis remains a major public health burden in India. Extrapulmonary tuberculosis (EPTB) commonly involves lymph nodes, most frequently affecting cervical regions. However, involvement of periportal or peripancreatic lymph nodes leading to obstructive jaundice is exceptionally rare, with only a limited number of cases reported globally [1].

In patients receiving anti-tubercular therapy (ATT), jaundice is most frequently attributed to drug-induced hepatotoxicity. However, the presence of cholestatic jaundice with radiological evidence of biliary obstruction warrants evaluation for alternative etiologies, including tuberculous lymphadenitis causing external compression of the biliary tree. Periportal lymphadenopathy can result in obstructive jaundice through extrinsic compression of the common bile duct (CBD), leading to upstream biliary dilatation.

This presentation poses a significant diagnostic challenge, as it may mimic malignancy or drug-induced hepatotoxicity in patients undergoing ATT. We present a rare case of multidrug-resistant (MDR) lymph node tuberculosis causing obstructive jaundice in a young male, highlighting the associated diagnostic challenges, the "rise and fall phenomenon," and the importance of appropriate interventional and pharmacological management. The "rise and fall phenomenon" refers to an initial clinical response to therapy, followed by subsequent deterioration due to underlying drug resistance.

## Case presentation

A 17-year-old male, resident of Nagpur, Maharashtra, India, a known case of MDR cervical lymph node tuberculosis on an all-oral longer MDR regimen, presented with gradually progressive yellowish discoloration of the eyes for 10-15 days, accompanied by pruritus, dark urine, and pale stools. The patient initially developed jaundice, followed by associated symptoms, while dull-aching right upper quadrant abdominal pain developed subsequently over the next five to six days, prompting further evaluation. There was no history of fever, vomiting, cough, hemoptysis, or weight loss.

On examination, the patient had icterus, with no clinical signs of hepatosplenomegaly or chronic liver disease. He also had a prior history of abdominal tuberculosis, for which he had received treatment for nine months.

Drug-induced hepatitis secondary to ATT was initially suspected, given its known association with ATT. However, the biochemical profile was more suggestive of a cholestatic pattern of liver injury rather than a hepatocellular pattern (Table 1). Viral hepatitis was considered as an alternative etiology but was excluded based on negative serological testing.

**Table 1:** Laboratory investigations

Lab Investigations	Findings	Reference range
Total bilirubin (mg/dL)	15.1	0.3-1.2
Direct bilirubin (mg/dL)	11.9	0.0-0.3
Indirect bilirubin (mg/dL)	3.2	0.2-0.9
Alkaline phosphatase (IU/L)	726	44-147
Viral hepatitis panel	Negative	

Differential diagnoses included drug-induced hepatotoxicity, cholangiocarcinoma, and obstructive pathology secondary to lymph nodal enlargement. Cholangiocarcinoma was considered less likely given the patient's young age and imaging features suggestive of a nodal mass rather than a primary biliary malignancy. Tuberculous lymphadenitis causing biliary obstruction was strongly suspected, supported by the clinical and radiological findings.

Histopathological confirmation was considered; however, due to the deep periportal location of the lesion and associated procedural risks, invasive biopsy was not pursued. The diagnosis was established based on clinical, biochemical, and radiological correlation.

The biochemical derangements indicated a cholestatic pattern of jaundice.

Imaging findings

**Figure 1:**
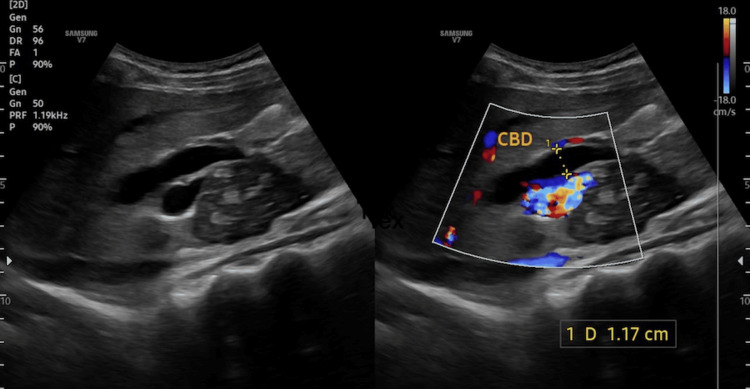
Transabdominal ultrasonography of the hepatobiliary system USG demonstrates dilatation of the common bile duct (CBD), measuring approximately 1.17 cm in diameter. The duct appears prominently dilated with evidence of upstream biliary dilatation. Color Doppler imaging shows no significant internal vascularity within the duct, supporting a non-vascular obstructive etiology.

Ultrasonography performed on 15/04/25 revealed hepatomegaly, with a liver span of 17.5 cm, along with dilated intrahepatic biliary radicals. The CBD and common hepatic duct (CHD) were also dilated, measuring 1.8 cm and 1.2 cm, respectively. A heterogeneous mass measuring 7 × 3.5 × 5 cm was identified adjacent to the pancreatic head and uncinate process (Figure 1).

**Figure 2:**
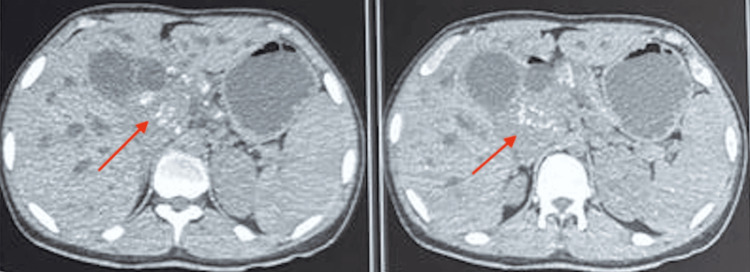
Contrast-enhanced CT (CECT) abdomen showing a periportal lymph nodal mass Axial CECT images of the abdomen demonstrate a heterogeneous, enhancing soft-tissue mass in the periportal region (red arrows). The lesion is seen encasing and compressing the common bile duct, resulting in upstream biliary dilatation.

Contrast-enhanced computed tomography (CECT) of the abdomen on 16/04/25 demonstrated a progressively enhancing periampullary and periportal mass, causing an abrupt, smooth cut-off of the CBD with significant upstream biliary dilatation (Figure 2). Based on imaging characteristics, the findings were suggestive of a tuberculous lymph nodal mass, and cholangiocarcinoma was considered unlikely.

**Figure 3:**
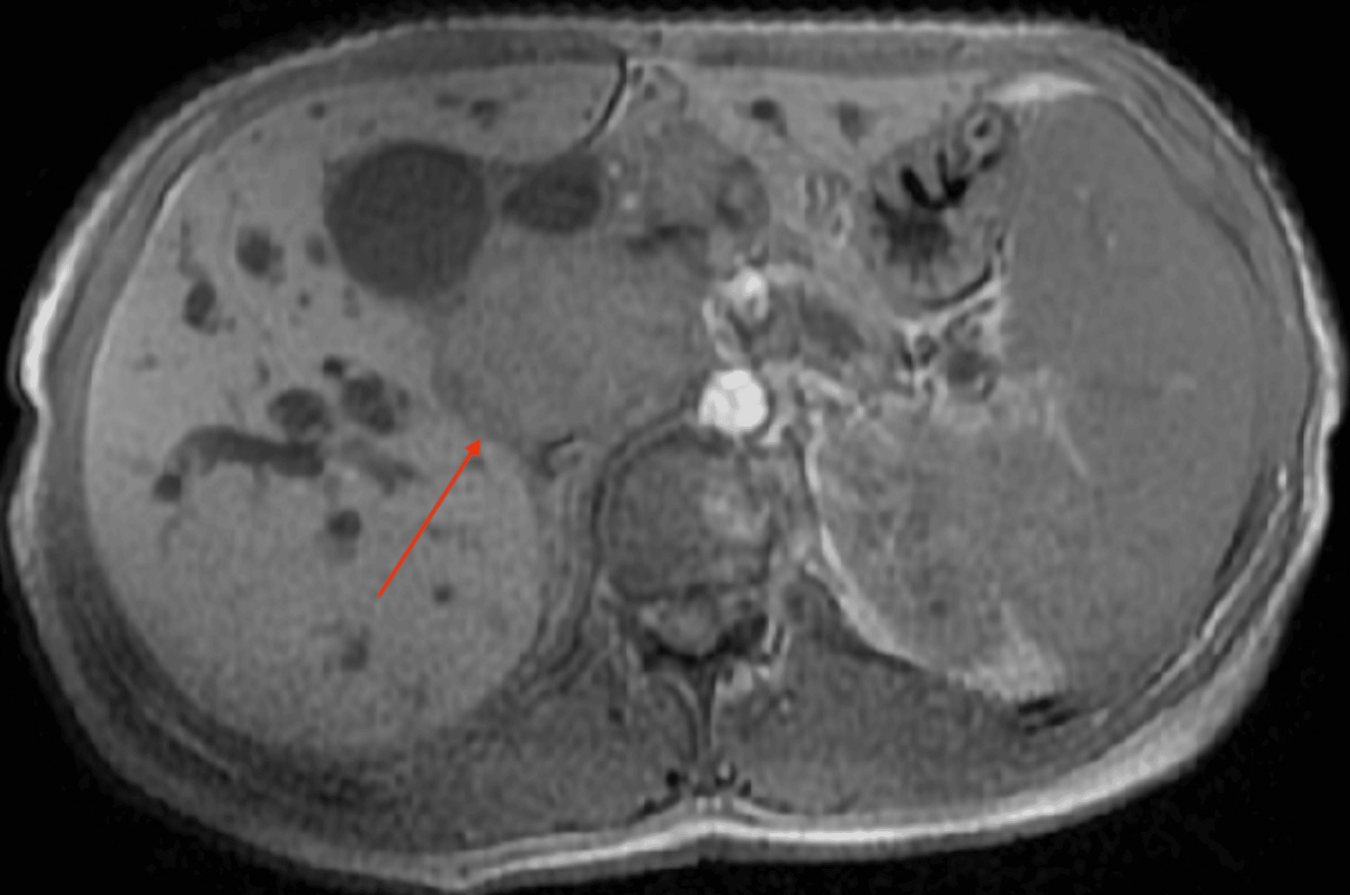
T1-weighted MRCP image showing a periportal lymph nodal mass Axial T1-weighted MR image demonstrates a hypointense conglomerate lymph nodal mass in the periportal and peripancreatic region causing extrinsic compression of the common bile duct.

**Figure 4:**
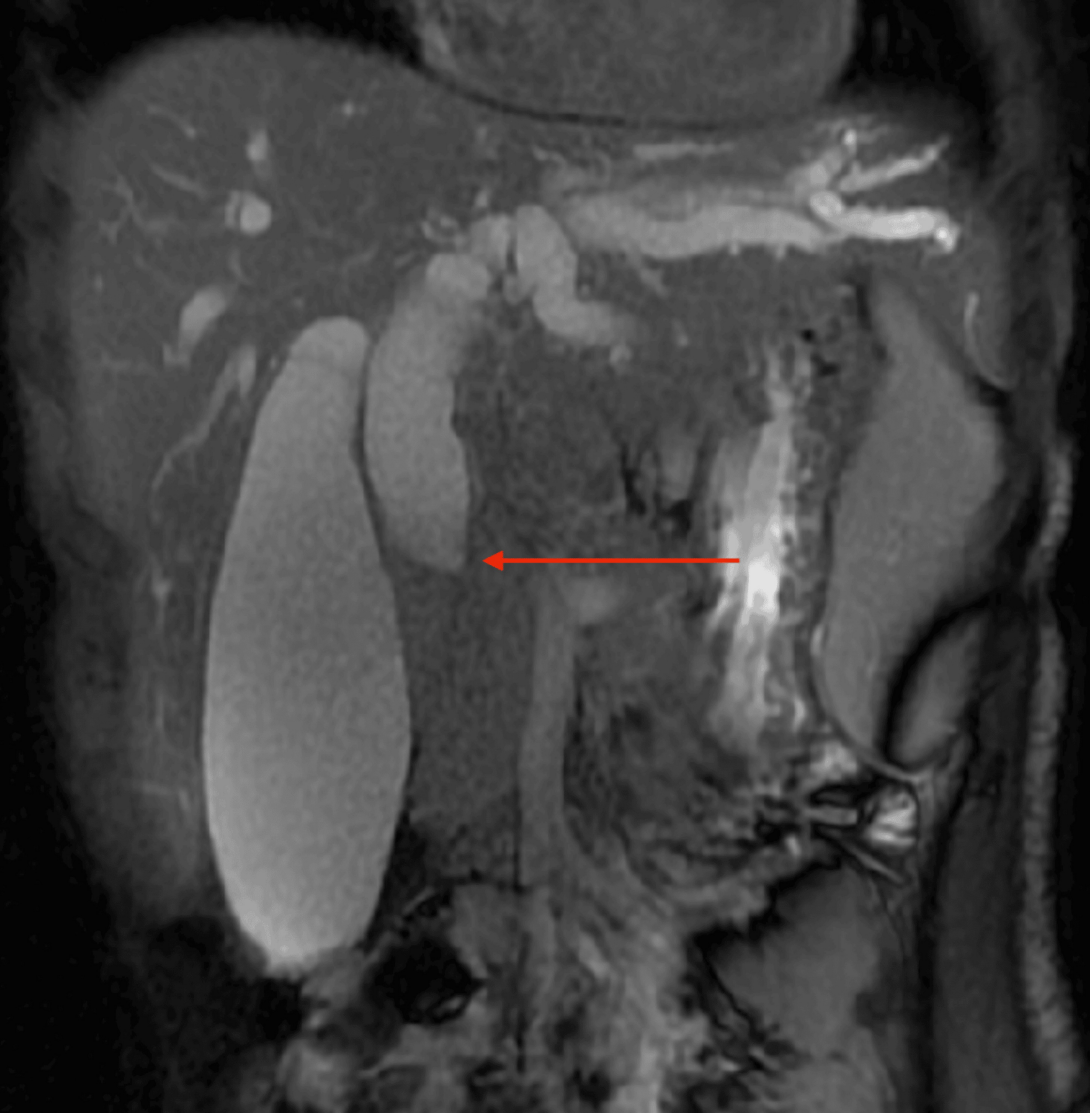
MRCP showing biliary obstruction due to a periportal lymph nodal mass Coronal MRCP image demonstrates a distended gallbladder and dilated intrahepatic biliary radicals with prominence of the common bile duct proximally. There is an abrupt narrowing at the mid common bile duct (red arrow), consistent with extrinsic compression. MRCP: magnetic resonance cholangiopancreatography

Magnetic resonance cholangiopancreatography (MRCP) performed on 21/04/25 showed a coalescent lymph nodal mass involving the periportal, pericoeliac, and peripancreatic regions. This mass was compressing the mid-CBD, resulting in upstream biliary dilatation (Figures 3-4).

Overall, the imaging findings were consistent with obstructive jaundice secondary to lymph nodal tuberculosis.

Management

The diagnosis was confirmed after all laboratory and radiological investigations. ATT was temporarily withheld in view of severe cholestasis. Management of tuberculous lymphadenitis causing obstructive jaundice was started. The patient underwent percutaneous transhepatic biliary drainage (PTBD) to relieve CBD obstruction, resulting in rapid symptomatic improvement and reduction in bilirubin levels. Total bilirubin decreased from 15.1 mg/dL to 4.1 mg/dL following PTBD, with corresponding improvement in liver enzymes, indicating effective biliary decompression. Supportive therapy included ursodeoxycholic acid, hydration, and nutritional management.

After stabilization, the patient was restarted on an all-oral longer MDR regimen with careful monitoring of liver enzymes.

Follow-up ultrasonography (21/08/25) revealed a reduction in lymph node mass from approximately 7 × 3.5 cm to 3.5 × 5 cm, along with improvement in biliary dilatation.

## Discussion

Tuberculous lymphadenitis causing obstructive jaundice is an extremely rare presentation. Obstructive jaundice due to tuberculous lymphadenitis typically results from external compression of the biliary tree by enlarged lymph nodes or associated mass lesions [2]. Alvarez found that, clinically and radiologically, such cases may mimic cholangiocarcinoma or pancreatic malignancy, especially in the presence of coalescent nodal masses [3].

Hepatobiliary tuberculosis develops through two principal pathways. One involves direct extension of caseating material from the portal tracts into the biliary ducts. The other occurs due to secondary inflammatory changes arising from tuberculous involvement of periportal lymph nodes (periportal adenitis) [4].

Drug-induced hepatotoxicity is the most common cause of jaundice in TB patients on ATT. However, cholestatic patterns of injury should prompt evaluation for obstructive causes. The present case also exhibited the "rise and fall phenomenon," wherein partial initial response to first-line therapy is followed by clinical deterioration due to underlying drug resistance [5].

For management of this condition, medical therapy alone is preferred; however, challenges remain: rising MDR *Mycobacterium tuberculosis *strains and recurrent inflammation causing severe bile duct injury, leading to permanent scarring and functional impairment [6].

In this case, PTBD was performed to relieve the tight obstruction of the CBD duct. PTBD was an effective and minimally invasive option, along with treatment of MDR-TB using a modified regimen, which was continued post-procedure for disease control.

This case shows the importance of multidisciplinary management involving pulmonologists, gastroenterologists, and interventional radiologists.

The diagnosis was supported by the presence of a cholestatic pattern of liver injury, radiological evidence of extrinsic biliary compression by a lymph nodal mass, and clinical improvement following biliary decompression and continuation of ATT. Alternative diagnoses, including drug-induced hepatotoxicity and malignancy, were considered less likely based on these findings.

## Conclusions

Obstructive jaundice in tuberculosis patients should not be automatically attributed to drug-induced hepatitis. Tuberculous lymphadenitis at the porta hepatis, although rare, can cause significant biliary obstruction. Early imaging, timely decompression with PTBD, and tailored MDR tuberculosis therapy are crucial for optimal outcomes. Early recognition prevents unnecessary discontinuation of ATT and avoids misdiagnosis as malignancy.
